# Lupus autoantibodies act as positive allosteric modulators at GluN2A-containing NMDA receptors and impair spatial memory

**DOI:** 10.1038/s41467-020-15224-w

**Published:** 2020-03-16

**Authors:** Kelvin Chan, Jacquelyn Nestor, Tomás S. Huerta, Noele Certain, Gabrielle Moody, Czeslawa Kowal, Patricio T. Huerta, Bruce T. Volpe, Betty Diamond, Lonnie P. Wollmuth

**Affiliations:** 1 Graduate Program in Neuroscience, Stony Brook University, Stony Brook, NY 11794-5230 USA; 2 Medical Scientist Training Program (MSTP), Stony Brook University, Stony Brook, NY 11794-5230 USA; 3 Department of Neurobiology & Behavior, Stony Brook University, Stony Brook, NY 11794-5230 USA; 4 Donald & Barbara Zucker School of Medicine, Hofstra University, Hempstead, NY 11549 USA; 5 Center for Autoimmune, Musculoskeletal and Hematopoietic Diseases, Feinstein Institute for Medical Research, Northwell Health, Manhasset, NY 11030 USA; 6 Graduate Program in Molecular and Cellular Pharmacology, Stony Brook University, Stony Brook, NY 11794-5230 USA; 7 Center for Biomedical Science, Feinstein Institute for Medical Research, Northwell Health, Manhasset, NY 11030 USA; 8 Department of Biochemistry & Cell Biology, Stony Brook University, Stony Brook, NY 11794-5230 USA; 9 Center for Nervous System Disorders, Stony Brook University, Stony Brook, NY 11794-5230 USA

**Keywords:** Diseases of the nervous system, Ion channels in the nervous system, Neuroimmunology, Systemic lupus erythematosus

## Abstract

Patients with Systemic lupus erythematosus (SLE) experience various peripheral and central nervous system manifestations including spatial memory impairment. A subset of autoantibodies (DNRAbs) cross-react with the GluN2A and GluN2B subunits of the NMDA receptor (NMDAR). We find that these DNRAbs act as positive allosteric modulators on NMDARs with GluN2A-containing NMDARs, even those containing a single GluN2A subunit, exhibiting a much greater sensitivity to DNRAbs than those with exclusively GluN2B. Accordingly, GluN2A-specific antagonists provide greater protection from DNRAb-mediated neuronal cell death than GluN2B antagonists. Using transgenic mice to perturb expression of either GluN2A or GluN2B in vivo, we find that DNRAb-mediated disruption of spatial memory characterized by early neuronal cell death and subsequent microglia-dependent pathologies requires GluN2A-containing NMDARs. Our results indicate that GluN2A-specific antagonists or negative allosteric modulators are strong candidates to treat SLE patients with nervous system dysfunction.

## Abstract (web summary)

Systemic lupus erythematosus (SLE) is an autoimmune disorder which can have neurological manifestations, including autoantibody targeting of the NMDA receptor. In this study, the authors GluN2A subunit is a target of SLE autoantibodies, using sample derived from patient.

## Introduction

Systemic lupus erythematosus (SLE) is an autoimmune disease characterized by the presence of autoantibodies directed against multiple self-antigens, including DNA^1^. These autoantibodies affect multiple organ systems such that SLE patients experience arthritis, renal disease, anemia, rashes, and neuropsychiatric symptoms, including memory disorders and spatial memory impairment^2–4^. The prevalence of diffuse nervous system disorders is reported from 20–90%, depending on the particular functional assessment^5–7^. These cognitive defects in both clinical and pre-clinical conditions are often associated with DNRAb, anti-double-stranded DNA (dsDNA) antibodies with cross-reactivity to NMDA receptors (NMDAR)^8–14^.

The role of DNRAbs in contributing to neuropsychiatric symptoms in SLE have largely been studied in mice models that endogenously synthesize DNRAbs and in mice exposed to patient-derived DNRAbs^8,10,11^. Patient-derived DNRAbs are IgG1 antibodies cloned from patient B cells that display reactivity to dsDNA and NMDARs^15,16^. Specific regions of the brain in mice are targeted based on the experimental method used to trigger blood brain barrier permeability—lipopolysaccharide (LPS) causes DNRAbs to deposit in the hippocampus while epinephrine causes DNRAbs to deposit in the amygdala^11,17^. Non-invasive imaging of SLE patients have revealed hippocampal atrophy and parahippocampal microstructural defects, conferring an advantage to using LPS in disease models^4,18^.

LPS-treated mice immunized to generate DNRAbs and patient-derived DNRAbs display a gamut of pathologies in the hippocampus: aberrant excitatory signaling, apoptosis, dendritic pruning, and microglial activation^10,11,19^. These mice also display expanded place fields in the hippocampus and defects in spatial memory^2,19^. These studies are essential to defining the pathology of the neuropsychiatric component of SLE, but do not define the specific NMDARs that mediate these effects. This information is critical to potentially develop therapies to treat and prevent neuropsychiatric symptoms associated with DNRAbs.

NMDARs are ionotropic glutamate receptors that are central to excitatory synaptic transmission in the brain. NMDARs are heterotetramers composed of two obligate GluN1 subunits and typically two GluN2 subunits of the same or different subtype (GluN2A, B, C, or D)^20,21^. DNRAbs bind to both GluN2A and GluN2B subunits, with the epitope including a pentapeptide consensus sequence, DWEYS^10,11^. This antigenic target for DNRAbs is in the extracellularly located amino-terminal domain (ATD) of GluN2, an allosteric hub for modulating NMDAR function^21,22^. NMDARs containing GluN2A or GluN2B have distinct physiological, pharmacological, and signaling properties^22^. Nevertheless, the contribution of the different GluN2 subunits to the SLE-associated neuropathologies is unclear. Knockout studies in mice have suggested GluN2A to be the primary target for DNRAb-mediated adult and fetal neuronal cell death, though the evidence was limited^23^. Specific inhibitors of GluN2B also reduced DNRAb-mediated cell death, suggesting a significant contribution of GluN2B to the DNRAb-mediated phenotype^10^. All these results are ambiguous because of uncertainty of antibody concentrations in vivo and variations of DNRAb preparations used in different passive transfer experiments.

Here, we use a combination of heterologous expression systems and animal models to show subunit-specific susceptibility to DNRAb-mediated pathological effects. For heterologous expression, we tested a DNRAb (G11 and its B1 isotype control) derived from a human patient (see “Methods”) in concentrations relevant to patient CSF levels^10^. We find that DNRAbs act as positive allosteric modulators (PAMs) at both GluN2A- and GluN2B-containing NMDARs, but that GluN2A-containing receptors have a much higher intrinsic sensitivity to DNRAb-mediated potentiation than GluN2B-containing NMDARs. Using a heterologous expression system to express triheteromeric GluN1/GluN2A/GluN2B, we find that a single GluN2A subunit confers high sensitivity to DNRAbs. We find that mice with a forebrain deletion of the GluN2B subunit display the full spectrum of DNRAb-mediated pathology, including acute loss of hippocampal CA1 neurons, dendritic abnormalities in surviving neurons, microglia activation, defective place cell fields, and impaired spatial memory. Conversely, GluN2A knockout mice are protected from the effects of DNRAbs. Thus, our work identifies the GluN2A subunit as the central mediator of NMDAR-associated nervous system pathology in SLE, supporting the use of GluN2A-specific negative allosteric modulators to treat SLE patients with brain dysfunction.

## Results

### DNRAbs preferentially potentiate GluN2A-containing NMDARs

**Fig. 1:**
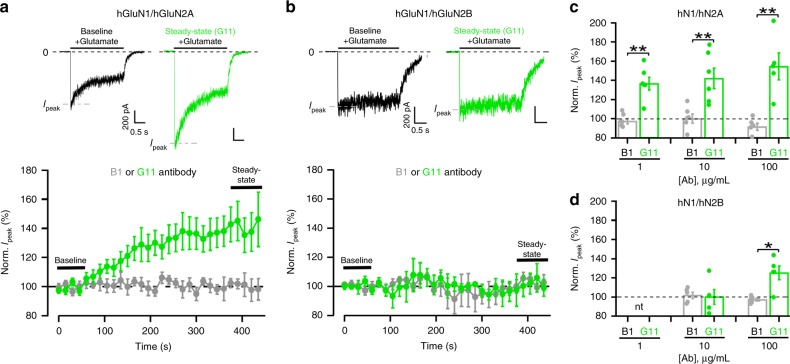
Differential sensitivity of N2A- and N2B-containing NMDARs to DNRAbs. **a**, **b** Direct DNRAb application to NMDARs. Upper panels, whole-cell currents from HEK293 cells expressing human NMDAR subunits, either hGluN1/hGluN2A (**a**) or hGluN1/hGluN2B (**b**) at 10 µg/mL. Currents were elicited by a 2.5 s application of glutamate (1 mM) in the continuous presence of glycine (0.1 mM) (holding potential, −70 mV). Lower panels, control antibody B1 (IgG1, gray circles) or human-derived DNRAb G11 (green circles) were added 75 s after a baseline recording of five sweeps, and were included in the bath throughout the remaining period. Current amplitudes for individual recordings were normalized to its baseline. Values are mean ± SEM (hN2A + B1, *n* = 6; hN2A + G11, *n* = 6; hN2B + B1, *n* = 5; hN2B + G11, *n* = 5; *n* = cells recorded). Example traces in upper panels show the +G11 recordings for the initial sweep during baseline (no antibody present) or for the last sweep during steady-state (in antibody). Ca^2+^ is not present in the extracellular solution to minimize run-down over time. **c**, **d** Peak current amplitudes in N2A-containing NMDAR are more strongly potentiated than those in N2B-containing receptors. Bar graphs (mean ± SEM with dots indicating individual values) (from left to right for hN1/hN2A, *n* = 6, 6, 6, 6, 5, 5 cells recorded; and for hN1/hN2B, *n* = 5, 5, 6, 5 cells recorded) showing normalized steady-state peak current amplitudes either for control antibody (B1) or DNRAbs (G11). Significance of DNRAb values are measured relative to their respective control (*p < 0.05 or **p < 0.01*,* two-sided *t* test) (left to right for N2A, *p* = 0.00137, 0.00932, 0.00906; left to right for N2B, *p* = 0.855, 0.0163). nt, not tested.

DNRAbs bind to both GluN2A and GluN2B subunits^2,10^. To begin to address how these subunits contribute to DNRAb-induced phenotypes, we characterized the effect of a SLE patient-derived monoclonal DNRAb, G11, on heterologously expressed NMDARs composed of human NMDAR subunits, either hGluN1-1a/hGluN2A (hN1/hN2A) or hGluN1-1a/hGluN2B (hN1/hN2B). In parallel with antibody titers found in SLE patient CSF samples, we test the DNRAbs at concentrations from 1–100 μg/ml^10^. For macroscopic or whole-cell currents, exposure of hN1/hN2A NMDARs to 10 μg/ml of G11 (green traces) strongly potentiated glutamate-gated current amplitudes compared with baseline (Fig. 1a). In contrast, a similar exposure to N2B-containing receptors had no effect on current amplitudes (Fig. 1b). A matched concentration of an isotype control antibody, B1 (gray traces), had no effect on current amplitudes for either N2A- or N2B-containing receptors.

For N2A-containing receptors, current amplitudes showed significant potentiation even at 1 μg/ml compared with its matched control (Fig. 1c). In contrast, only at 100 μg/ml, did N2B-containing receptors show significant potentiation relative to its control (Fig. 1d). Overall, these results suggest that, in terms of receptor gating, N2A-containing receptors are about 100-fold more sensitive to DNRAbs than N2B-containing receptors.

DNRAbs might potentiate NMDAR activity by acting as an agonist at the glutamate ligand-binding domain. To test this idea, we measured leak currents in the absence of glutamate in parallel to peak current amplitudes (Supplementary Table 1), but found that changes in leak current over time were not different in the presence of DNRAbs and their matched control. Hence, DNRAbs by themselves do not act as NMDAR agonists.

### DNRAbs act as positive allosteric modulators (PAMs)

**Fig. 2:**
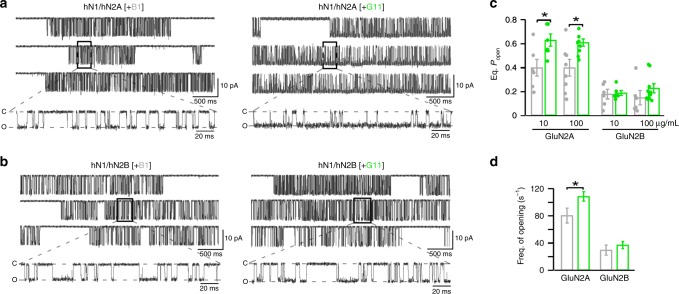
Single-channel recordings of N2A- and N2B-containing NMDARs under continuous DNRAb exposure. **a**, **b** Example single-channel recordings of hGluN1/hGluN2A (**a**) and hGluN1/hGluN2B (**b**) with B1 (left traces) or G11 (right traces) antibodies at 10 μg/ml. Recordings were made in the on-cell configuration (holding potential, +100 mV). Downward deflections are inward currents. Low resolution top traces show 12 s of recording (filtered at 1 kHz); more high-resolution bottom traces (selection in black box) show 230 ms (filtered at 3 kHz). **c** Equilibrium open probability (Eq. *P*_open_) (mean ± SEM) for N2A- or N2B-containing NMDARs (from left to right for hN1/nN2A, *n* = 6, 7, 8, 8 cells recorded; for hN1/hN2B, *n* = 6, 6, 6, 10 cells recorded). Antibody was either B1 (gray) or G11 (green) (*p < 0.05, two-sided *t* test) (left to right for N2A, *p* = 0.0335, 0.0226; left to right for N2B, *p* = 0.745, 0.0556). **d** DNRAbs enhance forward rates of activation. Frequency of single-channel openings (mean ± SEM) derived from samples in **c** with antibody either B1 (gray) or G11 (green) (*p < 0.05, one-sided *t* test) (left to right, *p* = 0.0270, 0.212). Test DNRAbs was 100 μg/ml.

To further verify these actions of DNRAbs, we measured single-channel activity of N2A- or N2B-containing NMDARs exposed either to control antibody B1 or to G11 (Fig. 2a–d). These recording were made in the on-cell configuration in the continual presence of glutamate and glycine (as well as 0.05 mM EDTA)^24^. The equilibrium open probability (eq. *P*_open_), an index of the ease of ion channel opening, with control antibody for wild-type hN1/hN2A (0.40 ± 0.07, *n* = 6) (mean ± SEM, *n* = cells recorded) and hN1/hN2B (0.18 ± 0.04, *n* = 6) is comparable to previously published values^25^. At 10 μg/ml of G11, hN1/hN2A single-channel activity was significantly potentiated (Fig. 2a) relative to the matched control (Fig. 2c). For hN1/hN2B, we again saw no significant effect of G11 on receptor gating at 10 μg/ml (Fig. 2b, c). At 100 μg/ml (Fig. 2c), the weak potentiation was not significant reflecting in part the variability of single-channel recordings of N2B-containing receptors. These results further indicate that N2A-containing receptors are more sensitive to DNRAbs than N2B-containing receptors.

To define how DNRAbs potentiate activity, we characterized single-channel details. Mean open time (MOT) was not significantly altered for either N2A- or N2B-containing receptors, whereas mean closed time (MCT) was significantly reduced for N2A-containing receptors at the highest concentration tested (Supplementary Table 2). Further, the frequency of opening was significantly enhanced for N2A-containing receptors (Fig. 2d). Thus, DNRAbs act by enhancing forward rates to the open state rather than by stabilizing the open channel.

### A single copy of GluN2A confers high DNRAb sensitivity

**Fig. 3:**
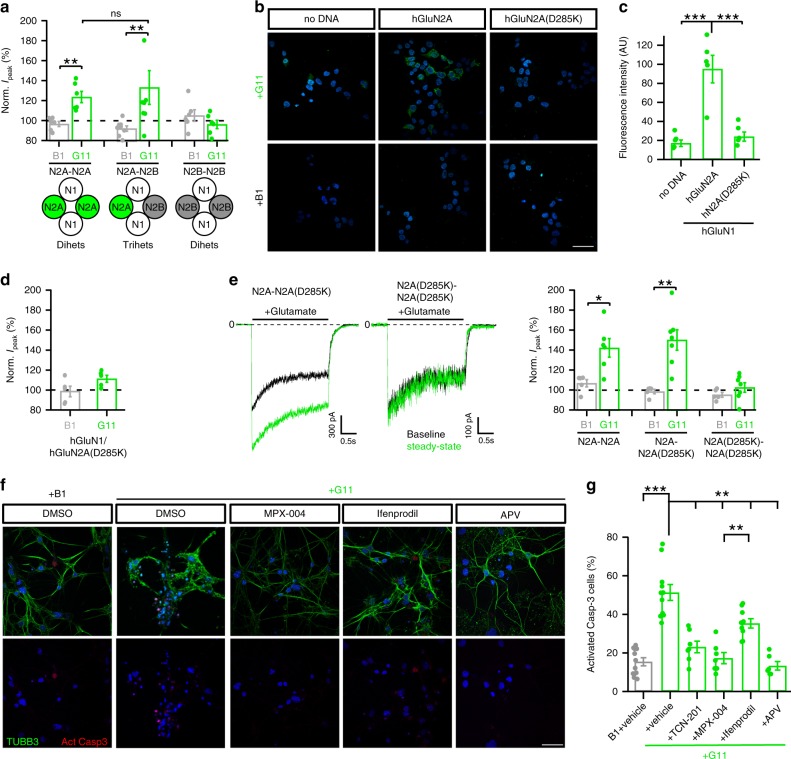
A single copy of GluN2A confers high sensitivity to SLE DNRAbs. **a** Bar graphs (mean ± SEM) (left to right, *n* = 6, 6, 8, 8, 6, 6 cells recorded) showing normalized whole-cell peak current amplitudes for diheteromeric (N1/N2A-N2A or N1/N2B-N2B) or triheteromeric (N1/N2A-N2B) receptors at 10 µg/mL of B1 or G11 antibody (**p < 0.01, two-sided Mann–Whitney *U* test) (left to right, *p* = 0.00507, 0.00865, 0.298; for ns, *p* = 0.581). **b** Representative images from immunocytochemistry of HEK293T cells not transfected (no DNA) or transfected with GluN1/GluN2A or GluN1/GluN2A (D285K), stained with B1 or G11 (10 μg/mL) (green, Alexa-488) and DAPI (blue) counterstain. Scale (white bar): 40 µm. **c** Mean fluorescence intensity in (B) (mean ± SEM) (*n* = 5 coverslips of cells, all conditions) (***p < 0.001, one-way ANOVA with post-hoc Tukey’s test) (Tukey’s: no DNA vs hN2A, *p* = 0.000156; hN2A vs hN2A(D285K), *p* = 0.000348). **d** Single charge reversal prevents DNRAb (10 μg/mL) potentiation of whole-cell currents (mean ± SEM, *n* = 5 recorded cells, all conditions). **e** Left, current records of triheteromeric (N1/N2A-N2A(D285K)) or diheteromeric (N1/N2A(D285K)-N2A(D285K)) NMDARs. Currents displayed as in Fig. 1a. Right, bar graphs (mean ± SEM) (left to right, *n* = 5, 6, 5, 7, 5, 6 recorded cells) showing normalized current amplitude for diheteromeric and triheteromeric receptors at 10 µg/mL of B1 or G11 (*p < 0.05, **p < 0.01, two-sided Mann–Whitney *U* test) (left to right, *p* = 0.0358, 0.00577, 0.194). **f** Immunocytochemistry of DIV14 primary hippocampal cultures incubated either in control (B1 + vehicle) or in DNRAb (G11) at 10 µg/mL. G11 was incubated either alone (+vehicle), with TCN-201 (not shown), MPX-004, or ifenprodil at 3 µM. Upper panels, representative images of neurons stained with antibodies against neuronal β-tubulin, Tubb3 (green, Alexa-488), and activated caspase-3 (red, Alexa-647), with DAPI (blue). Lower panel, only activated caspase-3 channel. Scale (white bar): 40 µm. **g** Quantification of immunocytochemistry shown in (**f**). Proportion of DAPI and activated caspase-3-positive cells divided by total DAPI cells (mean ± SEM) (left to right, *n* = 11, 11, 7, 7, 9, 6 coverslips of cells) per treatment condition (*p < 0.05,**p < 0.01, ***p < 0.001, one-way ANOVA with post-hoc Tukey’s test) (Tukey’s: G11 + vs G11 + MPX-004, *p* = 0.00282).

At native synapses, NMDARs are typically composed of GluN1 in combination with different GluN2 subunits, often one GluN2A and one GluN2B subunit, so-called triheteromeric receptors^26,27^. We therefore tested whole-cell currents from triheteromeric receptors, where the receptor contains a single copy of GluN2A (Fig. 3a). To generate triheteromeric receptors, we used NMDAR constructs having coiled–coiled domains that permit only specific subunit combinations to reach the plasma membrane^28,29^. At 10 μg/ml of DNRAb, diheteromeric N2A-containing receptors containing the coiled–coiled domains showed significant potentiation. Diheteromeric N2B-containing receptors again showed no potentiation. In contrast, triheteromeric receptors containing a single copy of GluN2A showed significant potentiation. Thus, a single copy of GluN2A confers high sensitivity to DNRAbs.

### DNRAbs modify receptor function via the DWEYS motif

DNRAb interact and presumably alter NMDAR function through the DWEYS motif in the amino-terminal domain. To directly test this idea, we introduced a charge reversal in the middle of the GluN2A DWEYS motif (DWDYS), mutating the negatively charged aspartate (D) at position 285 (DWDYS) to the positively charged lysine (K) (D285K). We avoided sites possibly involved in coordinating Zn^2+^ to mitigate confounding effects from Zn^2+^ modulation^30,31^. N2A(D285K) has no apparent effect on current properties (Supplementary Table 3). The G11 antibody shows robust binding to NMDARs (Fig. 3b, middle images). This binding is significantly attenuated in receptors containing D285K (Fig. 3b, right images, c). We did not find significant differences in the intrinsic binding of GluN2A or GluN2B-specific epitopes to G11 (Supplementary Fig. 1), suggesting that the difference in sensitivity primarily reflects gating. Receptors containing D285K are no longer significantly potentiated by DNRAbs (Fig. 3d), indicating that binding of DNRAbs to the DWEYS motif mediates the positive allosteric effect.

We also tested whether a single (triheteromeric receptor) or two (diheteromeric receptors) copies of the charge reversal are required to disrupt function. Consistent with the results for GluN2A/GluN2B triheteromeric receptors, a single copy of wild-type GluN2A confers full sensitivity to DNRAbs (Fig. 3e). Thus, in terms of stoichiometry, binding of a single DNRAbs to the DWEYS motif on GluN2A can generate the full positive allosteric effect.

### GluN2A antagonists are neuroprotective against DNRAbs

Our results indicate that N2A-containing receptors are significantly more sensitive to DNRAbs than N2B-containing receptors. To see if attenuating GluN2A activity is neuroprotective, we incubated primary hippocampal cultures either in control antibody (B1) (Fig. 3f, left images) or in G11 (10 μg/mL) in the absence or presence of GluN2A antagonists (TCN-201 or MPX-004), which are specific at the concentrations used here^28,32^, or a GluN2B-specific negative allosteric modulator (ifenprodil) and assayed the number of DAPI-positive cells associated with activated caspase-3 activity (Fig. 3g). In the presence of DNRAb alone, apoptotic cell death was extensive, consistent with previous results^3,8,33^. This cell death was significantly attenuated in the presence of MPX-004. In contrast, ifenoprodil was less able to protect against cell death (Fig. 3f, g). Thus, specific GluN2A antagonists are neuroprotective against DNRAb-mediated cell death.

### In vivo contributions of GluN2A and GluN2B to DNRAbs

**Fig. 4:**
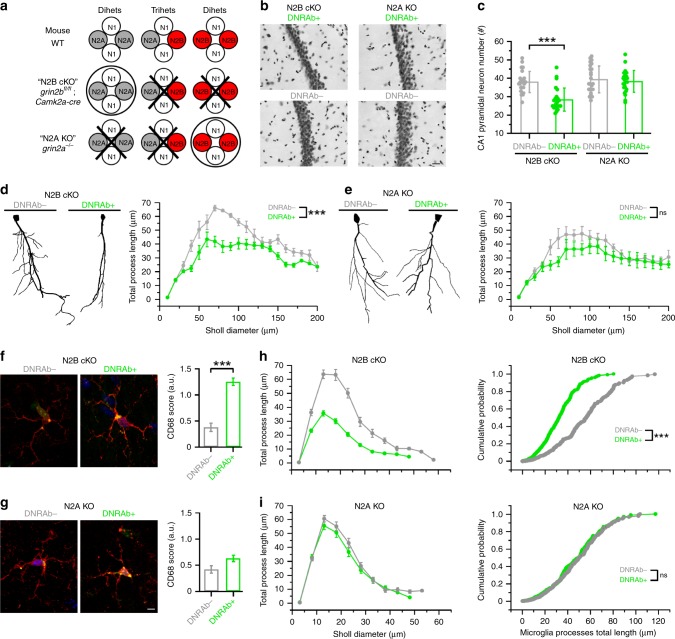
DNRAbs induced neuronal loss and dendritic pruning in CA1 pyramidal neurons persist in mice-lacking GluN2B, but not in those lacking GluN2A. **a** Schematic of NMDAR composition expected in hippocampal pyramidal neurons in the various genetic backgrounds (Supplementary Fig. 2). **b** Micrographs of hippocampal CA1 neurons from DNRAb+ or DNRAb− N2B cKO or N2A KO mice. Scale bar, 20 µm. **c** Quantification of CA1 neurons (mean ± SEM, *n* = 24 sections per group). Each dot represents a CA1 pyramidal neuron field counted using a standard unbiased stereological protocol (eight runs of systematic random sampling in three animals per group) (***p < 0.001, two-sided Mann–Whitney *U* test) (left to right, *p* = 0.0000156, 0.765). **d**, **e** Dendritic complexity of Golgi-stained neurons. Left, tracings of CA1 pyramidal neurons from N2B cKO (**d**) or N2A KO (**e**) mice. Right, Sholl analysis for N2B cKO (**d**) or N2A KO (**e**) mice (***p < 0.001, two-sided Kolmogorov–Smirnov test for data converted to cumulative distribution function) (left to right, p = 2.754 × 10^−16^, 0.61). Values at concentric rings are shown as mean ± SEM. For each group, 10–20 neurons per animal, 4 animals per group. **f**, **g** Representative images of microglia from N2B cKO (**a**) or N2A KO (**b**) mice. Microglia are labeled with antibody to Iba1 (red, Alexa 594) and to CD68 (green, Alexa-488). Scale (white bar): 5 µm. Right panels, quantification of CD68 + score (mean ± SEM) (***p < 0.001, Kruskal–Wallis ANOVA test) (left to right, *p* = 0.00000000222, 0.0793) (see Supplementary Methods). Three animals per group; number of quantified microglia: N2B cKO, DNRAb− (*n* = 42), DNRAb+ (*n* = 87), N2A KO, DNRAb− (*n* = 57), DNRAb+ (*n* = 58). **h**, **i** Microglia process complexity and length. Left panels, Sholl analysis of process length of microglia from N2B cKO (**h**) or N2A KO (**i**) mice. Right panels, cumulative probability distribution curves of microglia process length from N2B cKO (**h**) or N2A KO (**i**) (***p < 0.001, two-sided Kolmogorov–Smirnov test) (top to bottom, *p* = 7.16 × 10^−8^, 0.999). Three animals per group; number of quantified microglia: N2B cKO, DNRAb− (*n* = 33), DNRAb+ (*n* = 34); N2A KO, DNRAb− (*n* = 39), DNRAb+ (*n* = 40).

To address how the different NMDAR subunits contribute to the DNRAb-induced phenotypes in vivo, we used transgenic mouse models, either with the GluN2A subunit knocked out (*grin2A*^*−/−*^ mice, termed “N2A KO” henceforth) or with a conditional knockout of the GluN2B subunit (*grin2B*^fl/fl^*; CaMKII*^Cre^ mice, termed “N2B cKO”) (Fig. 4a). We used the conditional KO due to the embryonic lethality of a full GluN2B knockout^34–36^. KO mice were immunized with a decapeptide containing the pentapeptide, DWEYS, a mimetope of dsDNA and homologous to a sequence within the GluN2A and GluN2B extracellular domains, multimerized on a polylysine backbone (MAP-DWEYS) (Supplementary Fig. 2). Immunization of wild-type mice with MAP-DWEYS induces production of DNRAbs (DNRAb+ mice)^2,11,37^. As a control, mice were also immunized with the polylysine backbone alone (DNRAb− mice). Two weeks following two booster immunizations, mice were given LPS to allow transient access of antibodies to the hippocampus (Supplementary Fig. 2)^11,19^.

Wild-type mice, when immunized with MAP-DWEYS, show two stages of DNRAb-induced pathology (Supplementary Fig. 2)^2,11,19^. An acute phase, assayed one week after LPS treatment, that is characterized by extensive cell death of hippocampal pyramidal neurons^11^; and a chronic phase, assayed 8 weeks post-LPS treatment, characterized by the loss of dendritic complexity in pyramidal neurons, microglia activation, and disruption of place cell function (Supplementary Fig. 2). DNRAb+ mice also display reduced spatial memory, presumably mimicking the phenotype in SLE patients^2,11^. Because LPS only transiently permeabilizes the blood brain barrier^2^, the acute phase is associated with the presence of DNRAbs, whereas the chronic phase is independent of the direct presence of DNRAbs^2,19^.

### Mice lacking GluN2A are protected from DNRAb pathologies

Initially, we assayed the effect of DNRAbs on the acute phase of the pathology, namely the induction of pyramidal neuron cell death measured 1 week after LPS treatment^11,19^. For all in vivo experiments, we compare the effect in DNRAb+ mice to that in DNRAb− mice in the same genetic background with both of these groups treated with LPS (see Supplementary Fig. 2). DNRAb+ mice in the N2B cKO background, which still retain GluN2A, also displayed a significant loss of hippocampal neurons (Fig. 4b, c). In contrast, N2A KO mice showed no significant loss of neurons. Hence, the loss of pyramidal neurons during the acute phase is dependent on GluN2A.

Next, we characterized the chronic phase of pathology by assaying pyramidal neuron morphology 8 weeks after LPS administration (Fig. 4d–i). Compared with its control, GluN2B cKO mice exposed to DNRAbs exhibited a significant loss of dendritic complexity (Fig. 4d). In contrast, in GluN2A KO mice, DNRAbs have no significant effect on dendritic complexity (Fig. 4e). Thus, as with acute enhanced cell death, the chronic phase of SLE pathology associated with reduced dendritic complexity is also dependent on GluN2A.

### DNRAbs do not activate microglia in mice-lacking GluN2A

The reduced dendritic complexity occurring during the chronic phase of DNRAb-induced pathology is dependent on microglia activation^19^. We therefore characterized microglia in DNRAb+ and DNRAb− mice in the various genetic backgrounds (Fig. 4f–i).

For the N2B cKO background, and in comparison with its control, DNRAb+ mice showed enhanced expression of CD68 (Fig. 4f, right panel) and diminution of process complexity (Fig. 4h, left panel), and process length (Fig. 4h, right panel) over concentric Sholl diameters. These results are consistent with microglia activation and parallel what is observed in wild-type mice^19^. In contrast, for the N2A KO background (Fig. 4g–i), DNRAb+ mice, in comparison with its control, did not show significant CD68 expression (Fig. 4g, right panel) or reduced complexity (Fig. 4i), suggesting a lack or reduced microglia activation. Thus, DNRAb-induced microglia activation, which is critical in dendritic morphology changes, either directly requires GluN2A and/or is dependent on acute GluN2A-induced cell death.

### DNRAb-induced changes in spatial memory require GluN2A

**Fig. 5:**
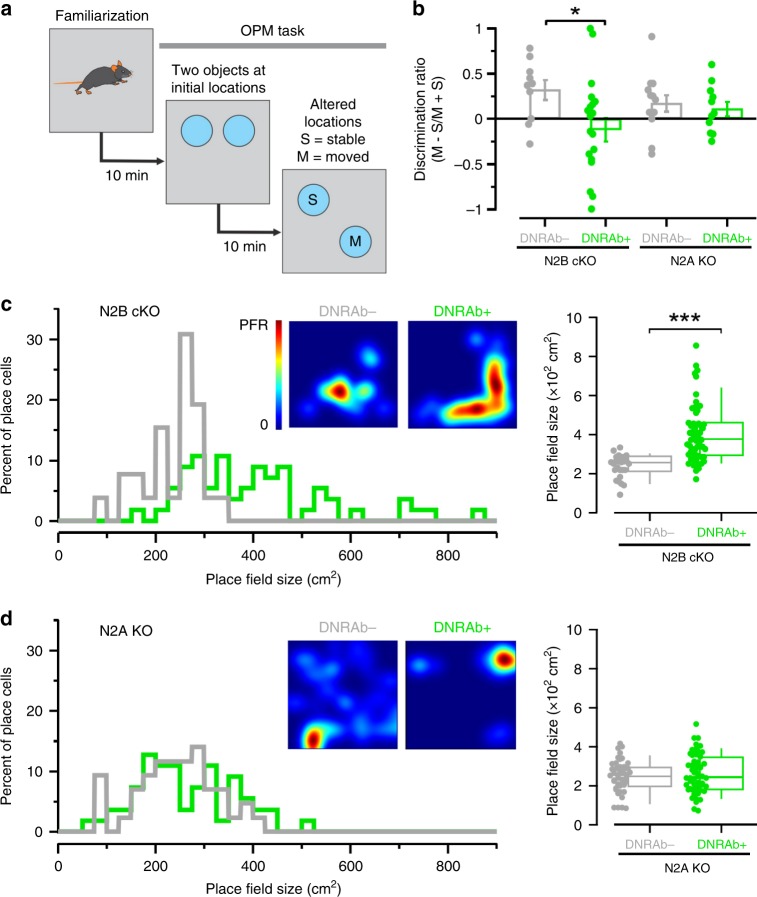
Behaviors disrupted by DNRAbs persist in mice-lacking GluN2B, but not in those lacking GluN2A. **a** Schematic of the object-place memory (OPM) task. **b** DNRAb+ N2B cKO mice show significantly reduced exploratory behavior, relative to its control, of the moved object over the stable object (Supplementary Fig. 2). Mice were tested 8 weeks after LPS treatment. Values shown are mean ± SEM (*n* = 10, 20, 13, 11, from left to right, number of tested mice per group; *p < 0.05, two-sided Mann–Whitney *U* test) (left to right, *p* = 0.0235, 0.524). **c**, **d** DNRAb disrupts place field size in mice-lacking GluN2B, but not in those lacking GluN2A. Left, histograms for place field sizes of all place cells recorded in DNRAb− (gray) or DNRAb+ (green) mice either N2B cKO (**c**) or N2A KO (**d**) backgrounds. Inset, heatmaps of representative place fields. Right, box plots (center: median; lower and upper box edge: 25th & 75th percentiles; with whiskers: 10th and 90th percentile), comparing place field size in N2B cKO (**c**) or N2A KO (**d**) mice (***p < 0.001, two-sided Mann–Whitney *U* test) (top to bottom, *p* = 0.0000000348, 0.670). Each dot represents a single place field: for N2B cKO (**c**), DNRAb− (*n* = 26) and DNRAb+ (*n* = 56); for N2A KO (**d**), DNRAb− (*n* = 43) and DNRAb+ (*n* = 55).

During the chronic phase of DNRAb-induced pathology, wild-type mice exposed to DNRAb show impaired spatial memory and disrupted place cell properties in the CA1 region of the hippocampus (Supplementary Fig. 2)^2,11,19^. We therefore asked whether DNRAb+ N2A KO and N2B cKO mice, 8 weeks after LPS exposure, behaved abnormally in an object-place memory (OPM) task, which tests spatial memory (Fig. 5a)^38,39^. N2B cKO mice transiently exposed to DNRAbs performed significantly worse than its DNRAb− control in the OPM task (Fig. 5b). This parallels to what is observed in wild-type mice (Supplementary Fig. 2). In contrast, no difference in performance was detected between DNRAb+ and DNRAb− N2A KO mice. Thus, GluN2A, either directly or indirectly, is required for DNRAb-induced disruptions in spatial memory.

NMDAR-dependent spatial memory and learning is linked to hippocampal place field size^40,41^. In wild-type mice exposed to DNRAbs, place field size is aberrantly enlarged, paralleling the reduced spatial memory in SLE patients and other neurodegenerative models^2,19,42^. To assess neuronal functional integrity, we measured place field size in freely moving mice by implanting tetrodes into the dorsal CA1 region (Fig. 5c, d). As in wild-type, DNRAb+ N2B cKO mice exhibited significantly larger place field sizes compared with DNRAb− N2B cKO mice (Fig. 5c), indicating that in the absence of GluN2B, the DNRAb-induced disruption of place field size persists. In contrast, DNRAb+ and DNRAb− N2A KO mice displayed no significant differences in place field size (Fig. 5d). Thus, the cognitive deficits associated with DNRAbs are intimately linked to GluN2A, but appear largely independent of GluN2B.

## Discussion

Our experiments demonstrate that the GluN2A NMDAR subunit is required for various neuropathologies associated with lupus autoantibodies. We find that DNRAbs act as positive allosteric modulators (PAM) on NMDARs with the GluN2A subunit showing a much higher sensitivity than the GluN2B subunit (Figs. 1 and 2). DNRAb exposure manifests in two distinct phases: first, synaptic disturbances and cell death with acute exposure, and second, a loss of dendritic complexity resulting from microglia activation that occurs even after antibody is no longer detected^10,19^. Presumably, the higher sensitivity of GluN2A to DNRAbs facilitates cell death during acute exposure. The chronic phase, including the cognitive and memory defects similar to what is seen in SLE patients^2,43^, are also dependent on GluN2A, but it is unknown whether it is a residual consequence of excitotoxicity encountered during the acute phase or a lasting change to NMDARs that predispose neurons to microglial-dependent pruning.

G11, the human DNRAb we tested, acts as a PAM on NMDARs via the DWEYS motif (Fig. 3b–d). DNRAbs are present in ~30–40% of SLE patients, with these DNRAbs being identified by binding to peptides containing the DWEYS motif^9,10,14,44^. Hence, we assume that the PAM action is a common feature of all DNRAbs. Consistent with this idea, polyclonal antibodies from the CSF of SLE patient with neuropsychiatric symptoms that bind the DWEYS motif induce NMDAR hyperfunction^12,33^. Nevertheless, it is possible that there are clonal variations in the magnitude of the PAM action as well as possible additional functional effects of DNRAbs, necessitating the study of more SLE patient DNRAbs.

The concentration of DNRAbs in the nervous system presumably varies during the pathophysiological course of SLE. At low concentrations, DNRAbs would affect exclusively GluN2A-containing receptors, while at high concentrations, DNRAbs would also affect GluN2B diheteromeric receptors (Fig. 1). Previous work has shown that the GluN2B-specific inhibitor, ifenprodil, can be neuroprotective^10^. However, this most likely reflects that triheteromeric GluN1/GluN2A/GluN2B NMDARs, which constitute a major portion of hippocampal synapses^26,27^, are inhibited by ifenprodil^28,29^. Indeed, we find that a single copy of wild-type GluN2A confers high sensitivity to DNRAbs (Fig. 3a), a result consistent with GluN2A dominance in allosteric modulation^45,46^.

The DWEYS motif is located in the amino-terminal domain (ATD) clamshell hinge region (Fig. 3b–d). GluN2A-selective PAMs and negative allosteric modulators (NAMs) have been developed to target NMDAR hypo- or hyperfunction in disease^47,48^. While most GluN2A-selective PAMs and NAMs target the ligand-binding domain (LBD), the ATD interacts with the LBD to mediate its allosteric action^49–52^. In GluN2A/GluN2B NMDARs, the GluN2A ATD interacts more extensively with the LBD than the GluN2B ATD^45,46^, which may underlie the higher sensitivity of GluN2A to DNRAbs. Furthermore, the DWEYS motif is proximal to binding sites for other ATD allosteric modulators. Indeed, the sensitivity to Zn^2+^, a NAM, may be modulated by SLE antibodies^33^. Zn^2+^ is coordinated by several residues in the ATD clamshell that are proximal to or at the DWEYS motif^30,31,53,54^. It may be that the DNRAb-induced potentiation and SLE pathology observed through GluN2A is influenced by the relief of tonic Zn^2+^ given the differences in Zn^2+^ sensitivity between GluN2A and GluN2B^31,55,56^. Nevertheless, the mechanism of action of DNRAbs to produce positive allosteric modulation and the differences in sensitivity between GluN2A and GluN2B remain unknown, but are critical to define for potential therapeutic interventions.

Our work here does not discount the possibility that DNRAbs act on additional mechanisms, independent of NMDAR gating, to exert their subunit-specific effects. The pathology of anti-NMDAR encephalitis, for example, largely does not involve receptor gating, but rather changes in cell biology: autoantibodies cause internalization of NMDARs and chronic NMDAR hypofunction^57,58^, displace the interaction of NMDAR with synaptic proteins such as the EphrinB2 receptor^59,60^ or disrupt the nanoscale organization of NMDARs at the synapse with subunit-selectivity^61^. DNRAbs may also engage different intracellular pathways associated with GluN2A and GluN2B. Interestingly, GluN2A signaling has been associated with cell survival through activation of the transcription factor CREB, leaving the mechanism of GluN2A-mediated potentiation to be somewhat perplexing in the context of potentiation from DNRAbs^62^. However, there is increasing evidence to suggest that GluN2A over-activation can contribute to excitotoxicity^62–64^. GluN2A contributes to both the acute and chronic phases of SLE neuropathology. While the use of GluN2A NAMs may be key to treating the neuropsychiatric symptoms of SLE, any NMDAR inhibitor may lead to undesirable side effects^20,65,66^. Uncovering the mechanistic details of DNRAb-NMDAR interactions during distinct pathophysiological phases will help us better develop and tailor therapeutic strategies for preventing neuropsychiatric symptoms in SLE patients before acute events occurs, and treating them during chronic phases of SLE.

## Methods

See Supplementary Information–Supplementary Methods for details.

### Animals

Mice (females, C57BL/6 strain) were housed at the Center for Comparative Physiology at the Feinstein Institute for Medical Research. All protocols were approved by the local institutional animal care and usage committee (IACUC). Mice with deletion of the GluN2A subunit (*Grin2a*^*−/−*^) were kindly provided by Dr. M. Mishina (University of Tokyo). Mice with conditional knockout of the GluN2B subunit (*Grin2b*^fl/fl^*;Camk2a-cre*) were bred in house by crossing homozygous floxed GluN2B mice (*Grin2b*^fl/fl^, kind gift from Prof. Dr. H. Monyer) with a Cre line driven by CaMKIIα promoter (*Camk2a*-cre mice, B6.Cg-Tg(Camk2a-Cre)T29-1Stl/J, stock no: 005359, Jackson labs). Both groups were further bred on an H2d^+/+^ background to allow antibody response to immunization. Mice are housed in a laboratory animal facility at a 12/12 h light/dark cycle, and received water and food ad libitum. Female mice aged 6–8 weeks were immunized with MAP-core and MAP-DWEYS^11^.

All animal procedures at Stony Brook University were approved by the institutional animal care and usage committee (IACUC) at Stony Brook University and were in accordance with the guidelines established by the National Institutes of Health. Mice are housed in a laboratory animal facility at a 12/12 h light/dark cycle, and received water and food ad libitum. Mice (C57BL/6) were bred in house for primary cultures of hippocampal neurons at P0–P1 and glia/astrocyte feeder layers at P2–P4. Pups were not sexed for generation of primary cultures.

### SLE antibodies

G11 is an IgG1 monoclonal antibody derived from a female SLE patient’s B cells with Igγ1 heavy chain and Igκ light chain composition. B1 is an isotype IgG1 control derived from the same patient^16^. G11 was cloned from a B-cell binding the DWEYS peptide and B1 from a B cell that did not bind the peptide or any brain antigen. The SLE patient from whom the antibodies were derived had serum antibodies reactive to dsDNA and the DWEYS peptide^2,4^.

### In vitro electrophysiology

Human embryonic kidney 293 (HEK293) cells were grown in Dulbecco’s modified Eagle’s medium (DMEM), supplemented with 10% FBS, for 24 h before transfection. Human NMDAR-encoding cDNA constructs, were co-transfected into HEK293 cells along with a separate peGFP-Cl construct at a ratio of 4:4:1 (N1:N2:eGFP) for macroscopic recordings, and at a ratio for 4:1.5:1 for single-channel recordings using X-tremeGene HP (Roche, 06-366) (see Supplementary Methods for construct tables). Transfection of the triheteromeric NMDA receptor constructions were done with Ca^2+^ precipitation^28,29^.

Macroscopic currents in the whole-cell mode were recorded at room temperature (20–23 °C) using an EPC-10 (HEKA) amplifier with PatchMaster software (version 2 × 90.2, HEKA), digitized at 10 kHz and low-pass filtered at 2.9 kHz (−3 dB) using an eight pole low-pass Bessel filter 4^67,68^. Patch microelectrodes were filled with an intracellular solution (mM): 140 KCl, 10 HEPES, 1 BAPTA, 4 Mg^2+^-ATP, 0.3 Na^+^-GTP, pH 7.3 (KOH), 297 mOsm (sucrose). Our standard extracellular solution consisted of (mM): 150 NaCl, 2.5 KCl, and 10 HEPES, pH 7.2 (NaOH). Pipettes had resistances of 2–6 MΩ when filled with the pipette solution and measured in the standard Na^+^ external solution. Ca^2+^ was omitted from the extracellular solution to prevent run-down over time. We did not use series resistance compensation nor did we correct for junction potentials. Currents were measured within 10 min of going whole cell. External solutions were applied using a piezo-driven double-barrel application system. Prior to use, we incubated the application system with 2% BSA (1xPBS) for two hours at 21–24 °C to minimize non-specific antibody binding. For agonist application, one barrel contained the external solution +0.1 mM glycine, whereas the other barrel contained both 0.1 mM glycine and 1 mM glutamate. For display, NMDAR currents were digitally refiltered at 500 Hz and resampled at 1 kHz.

Single-channel currents were recorded in the on-cell configuration at 20–23 °C using an integrating patch clamp amplifier (Axopatch 200B, Molecular Devices), analog filtered at 10 kHz (four-pole Bessel filter), and digitized between 25 and 50 kHz (ITC-16 interfaced with PatchMaster, HEKA)^24,69^. Patch pipettes (thick-wall, borosilicate, Sutter Instruments) were pulled and fire-polished achieving resistances between 10 and 20 MΩ when measured in the bath. At −100 mV, seal resistance ranged between 2 and 20 GΩ. For cell-attached recordings, patch pipettes were filled with the standard bath solution as well as 1 mM glutamate and 0.1 mM glycine. In total, 0.05 mM EDTA was added to minimize gating effects of divalents. Inward currents were elicited by applying a pipette potential of +100 mV. Recordings of hGluN1/hGluN2A or hGluN1/hGluN2B, either in the presence of absence of DNRAbs consisted of long clusters of activity separated by seconds-long periods of inactivity, simplifying detection of several channels in the patch. For these recordings, the relatively high equilibrium open probability (eq. Po) and duration of recordings (~10,000 to 180,000 events per recording) indicated that we were recording from single-channel patches.

### Immunocytochemistry (ICC)

HEK293T cells were used for ICC. Cells were fixed 48 h post-transfection, washed with 1× PBS, and then blocked with 2% bovine serum albumin (BSA) (Sigma, A9647). Incubation of primary antibody (20 µg/mL), either G11 or control B1, diluted in 2% BSA was done overnight at 4 °C.

Primary hippocampal neurons were made^70^ with minor modifications (see Supplementary Methods). Pharmacological treatment occurred at DIV14. Final concentrations of antagonists were 3 µM: TCN-201 (AdooQ Bioscience, A11947), MPX-004 (Alomone Labs, M280), and Ifenprodil (Sigma, I2892). ICC was performed 24-h post treatment on DIV15. Coverslips were washed with 1× PBS, fixed, and washed again with 1× PBS and blocked/permeabilized with antibody blocking solution, consisting of 2% (w/v) BSA, 0.25% (v/v) Triton-X100 in 1xPBS, for 60 min. Coverslips were then incubated in primary antibodies, rabbit anti-activated caspase-3 (1:250, Cell Signaling, 9661), and mouse anti-β tubulin III (Tubb3) (1:400, Millipore-Sigma, MAB1637), both in antibody blocking solution overnight in 4 °C. Coverslips were washed with 1xPBS, and incubated in fluorescence-conjugated secondary antibodies, Goat anti-rabbit Alexa-647 (1:1000, ThermoFisher, A21245) and Goat anti-mouse Alexa-488 (1:1000, ThermoFisher, A21121) for 1 h at RT and mounted as described above. Post-processing and image analysis are detailed in Supplementary Methods.

### Neuronal staining

Mice were anesthetized with 100 μl of Euthasol (Virbac) prior to perfusion with 0.9% sodium chloride, 0.5% sodium nitrite, and 0.1% heparin, followed by 4% paraformaldehyde (PFA) in 0.1 M phosphate buffer (PB) as before^19^.

For cresyl violet staining, brains were extracted, fixed in 4% PFA for 2 h and transferred to 30% sucrose, blocked and sliced at 40 µm with a periodicity of one in four coronal sections starting at approximately Bregma −0.94 mm over the next 1600–1900 µm. Sections were mounted, dehydrated, rehydrated, and stained in cresyl violet for 3 min. Sections were dried, dehydrated, cleared (Histoclear II), and coverslipped with Permount® (Thermo Fisher) prior to imaging on an AxiophotZ1 microscope (Zeiss). Sections were imaged in 100× oil, and we focused on the soma of the stratum pyramidale around which a tiled Z-stacked (0.5 µm steps) image was generated. Stereology was performed with the sampling frame set such that an individual frame captures 2–5 targets in the photomicrograph^11,19^. Optical dissectors are then created by manipulating the level of the Z-stack, and nuclei within neurons in focus are counted as long as they are not in contact with the left or bottom part of a frame. Quantitation of CA1 neurons in image stacks was performed with the Stereo Investigator suite in Neurolucida 360 (MBF Bioscience). Neuron numbers in (Fig. 4c) represent the total number of soma captured in the systematic random sampling frame as it is placed on consecutive sections or “runs”. Neuron numbers were measured per sampled tissue volume (0.20 mm^3^ NR2B cKO DNRAb+; 0.19 mm^3^ NR2B cKO DNRAb−; 0.20 mm^3^ NR2A KO DNRAb+; 0.21 mm^3^ NR2A KO DNRAb−, mean ± 0.001 mm^3^, s.d.). There were three animals in each group, with eight runs for each animal.

For Golgi staining, brains were processed using FD Rapid Golgi Stain kit (FD NeuroTechnologies), and sectioned at 100 µm on a cryostat (−21 °C) that permit identification of internal anatomic landmarks to signal sample start and stop, with periodicity of one in four coronal sections across ~1200 µm (Bregma −0.94 to −2.2 mm for the dorsal hippocampus) of brain with at least five different sections being sample per animal. Sections were mounted, dried (room temperature, dark), silver nitrate stained, rinsed, dehydrated in EtOH, cleared in xylene, and coverslipped. Tissue was imaged on an AxioImager Z1 microscope (Zeiss) in 40× oil using tiling and Z-stack (2 µm steps). There were four animals in each group, and 10–20 neurons were analyzed for each animal as shown in Fig. 4d, e. Images were then analyzed using Neurolucida 360 (MBF Bioscience) dendritic tracing for Sholl analysis at 10 µm shells from the soma^2,19^.

### Immunohistochemistry

Mice were anesthetized as described above for cresyl violet staining. Brains were extracted, fixed, and transferred to 30% sucrose, blocked and sliced (40 µm). Sections were dried, dehydrated, cleared (Histoclear II) and coverslipped with Permount® (ThermoFisher) prior to imaging on an AxiophotZ1 microscope (Zeiss). For immunohistochemistry used in labeling microglia in Fig. 4f, g, sections were washed with PBS, permeabilized, blocked, and stained with primary antibody overnight at 4 °C. Primary antibodies were Iba1 (1:500, Wako Chemicals, 019-1 9741), CD68 (1:500, Bio-Rad, mca1957). On the following day, samples were washed with PBS and then incubated with secondary antibody, which included Donkey anti-rat Alexa Fluor 488 (1:400, Life Technologies, A21208), Chicken anti-rabbit Alexa Fluor 594 (1:300, Life Technologies, A21442). Post-processing and image analysis are detailed in Supplementary Methods.

### Behavioral assessment and in vivo electrophysiology

Mice (20-24 weeks) were handled for 15 min per day for 3 days before being tested behaviorally. The object-place memory (OPM) task was performed^2,19^. The apparatus consisted of a square (40 cm) chamber 40 cm high, with the walls painted gray. Animals were familiarized to the empty chamber (three sessions of 15 min each). For OPM testing, mice underwent the following sequence: empty chamber (10 min), home cage (10 min), sample phase in which the chamber had two objects located at the center of the NW and NE sectors (5 min), home cage (10 min), choice phase, in which the chamber had the same objects but one of them was moved from NE to the center of the SE sector (5 min). The discrimination ratio was calculated during the choice phase by dividing time spent exploring the moved object minus the time spent exploring the static object by the time exploring the objects combined^2^. Data were collected and analyzed using EthoVision XT (version 11, Noldus Information Technologies).

In vivo recordings of place cells in the dorsal CA1 of the hippocampus were made^2,19^. Mice were anesthetized and implanted with a 16-channel multi-electrode array. After recovery, single unit firing was recorded using Cheetah software (version 5, Neuralynx), while the animal explored a square chamber in a schedule of four exploration runs separated by three rest sessions. Data were analyzed using Spike2 (version 8, Cambridge Electronic Design), NeuroExplorer (version 5, Nex Technologies), and MATLAB (version 9.2 R2017a, MathWorks).

### Statistics

Data analysis was performed using IgorPro (version 7, WaveMetrics), QuB (version 2.0.0.30, SUNY at Buffalo), Microsoft Excel, and ImageJ. All average values are presented as mean ± SEM. For statistical analysis, we used either Origin Pro (version 9, Origin Lab) or MiniTab (version 19, Trialware). Normality was used to determine appropriate statistical tests. For normally distributed data, one-tailed or two-tailed Student's *t* tests (*t* test) as indicated or one-way ANOVAs with post-hoc Tukey’s test were used as indicated. For non-normally distributed data or instances where the data were categorical variables or cumulative distribution functions, Mann–Whitney *U* tests, Kruskal–Wallis ANOVAs, and Kolmogorov–Smirnov tests were used as indicated. Significance was typically *p < 0.05.

We did not run a statistical test to determine sample size a priori. Sample sizes resemble those from previous publications.

### Reporting summary

Further information on research design is available in the Nature Research Reporting Summary linked to this article.

## Supplementary information

Supplementary Information

Reporting summary

## Data Availability

The data sets generated and analyzed during this study are available from the corresponding authors on reasonable request. The source data underlying Figs. 1–5 has been provided as a Source Data file.

## Source Data

Source Data

## Code availability

Code used for analysis is available from corresponding authors on reasonable request.
