# Supplementary material for: Lupus autoantibodies act as positive allosteric modulators at GluN2A-containing NMDA receptors and impair spatial memory
Source: Nat Commun. 2020 Mar 16;11:1403. doi: 10.1038/s41467-020-15224-w (PMC7075964; doi:10.1038/s41467-020-15224-w)
Supplement: Supplementary file 1 — Supplementary Information [file 41467_2020_15224_MOESM1_ESM.pdf]

SUPPLEMENTARY INFORMATION

Lupus autoantibodies act as positive allosteric modulators at GluN2A-containing NMDA receptors and impair spatial memory

Kelvin Chan, Jacquelyn Nestor, Tomás S. Huerta, Noele Certain, Gabrielle Moody, Czeslawa Kowal, Patricio T. Huerta, Bruce T. Volpe, Betty Diamond, and Lonnie P. Wollmuth

TABLE OF CONTENTS

Supplementary Table 1. In the whole-cell mode, the G11 antibody has no notable effect on current properties other than changes in peak current amplitudes (relates to Figures 1A-1D). 3

Supplementary Table 2. Effects of DNRAbs on hGluN2A- or hGluN2B-containing NMDARs (relates to Figures 2A-2D). 4

Supplementary Table 3. The D285K mutation in the GluN2A DWEYS motif has no notable effect on current properties (relates to Figures 3B-3D). 4

Supplementary Figure 1. Competitive ELISAs for DNRAb binding to soluble GluN2A or GluN2B epitopes (relates to Figures 3B-3E) 5

Supplementary Figure 2. Diagram of the experimental protocol used for using the NPSLE mouse model (relates to Figures 4 & 5) 6

Supplementary Methods 7

Supplementary Information References 12

Supplementary Table 1. In the whole-cell mode, the G11 antibody has no notable effect on current properties other than changes in peak current amplitudes (relates to Figures 1A-1D).

Subunit	Antibody	Norm. I_{peak} %	Norm. I_{leak} %	Norm. Des %	n
hGluN1/hGluN2A	100 µg/ml B1	92 ± 3.6	173 ± 44	105 ± 4	5
	100 µg/ml G11	**154* ± 14**	194 ± 95	73 ± 25	5
hGluN1/hGluN2B	100 µg/ml B1	97 ± 1.4	121 ± 12	121 ± 16	6
	100 µg/ml G11	**125* ± 7.2**	92.1 ± 13	103 ± 16	5

Values shown are mean ± SEM for changes in normalized whole-cell peak current amplitudes (Norm. I_{peak}), normalized leak current amplitudes (Norm. I_{leak}), and normalized extent desensitization (Norm. Des) for steady-state currents either in control antibody (B1) or human DNRAb (G11) (see Figures 1A & 1B).
Tagged values are significantly different (*) than their respective control condition, B1 (*$p < 0.05$, *two-tailed Student's t-test, unpaired*).

We made these measurements in 100 µg/ml where both N2A- and N2B-containing receptors were potentiated (Figures 1C & 1D). The lack of an effect on leak current indicates that DNRAbs do not themselves act as agonists. DNRAbs also do not affect desensitization.

Supplementary Table 2. Effects of DNRAbs on hGluN2A- or hGluN2B-containing NMDARs (relates to Figures 2A-2D).

Subunit	Antibody	Total events (# of patches)	*i* *pA*	eq. P_{open}	MCT *ms*	MOT *ms*
hGluN2A	10 µg/ml B1	485793 (6)	-8.3 ± 0.4	0.40 ± 0.07	5.9 ± 1.0	3.8 ± 0.6
	10 µg/ml G11	546303 (7)	-8.2 ± 0.5	**0.63* ± 0.05**	3.5 ± 0.7	6.0 ± 0.9
	100 µg/ml B1	632383 (8)	-7.1 ± 0.2	0.40 ± 0.07	8.9 ± 1.9	4.9 ± 0.7
	100 µg/ml G11	1047339 (8)	-7.5 ± 0.1	**0.61* ± 0.03**	**3.7* ± 0.4**	5.7 ± 0.4
hGluN2B	10 µg/ml B1	135134 (6)	-8.3 ± 0.3	0.18 ± 0.04	27.9 ± 12.4	4.0 ± 0.7
	10 µg/ml G11	445555 (6)	-8.5 ± 0.2	0.19 ± 0.02	16.0 ± 2.9	3.5 ± 0.3
	100 µg/ml B1	154900 (6)	-7.5 ± 0.4	0.15 ± 0.06	42.9 ± 12.9	4.8 ± 0.9
	100 µg/ml G11	332097 (10)	-7.5 ± 0.3	0.23 ± 0.04	24.0 ± 2.8	6.1 ± 0.5

Values shown are mean ± SEM for single-channel current amplitude (*i*), equilibrium open probability (P_o), mean closed time (MCT), and mean open time (MOT). Single channel currents were recorded in the on-cell mode at approximately -100 mV and analyzed in QuB (see Materials & Methods). Number of patches is in parenthesis to the right of total events. Eq P_o is the fractional occupancy of the open states in the entire single-channel recording, including long lived closed states (desensitized states). All data were idealized and fit at a dead time of 20 µs.
Tagged values are significantly different (*) than their respective isotype control antibody (Ab) condition, B1 (*$p < 0.05$, two-tailed Student's t-test, unpaired*).

Supplementary Table 3. The D285K mutation in the GluN2A DWEYS motif has no notable effect on current properties (relates to Figures 3B-3D).

Subunit	I_{ampl} *pA*	Des *%*	n
hGluN1/hGluN2A	-1200 ± 550	33 ± 12	10
hGluN1/ hGluN2A(D285K)/hGluN2A(D285K)	-970 ± 290	19 ± 10	7

Values shown are mean ± SEM for whole-cell peak current amplitude (I_{ampl}) and % desensitization (Des). Wild type and D285K recordings were alternated.

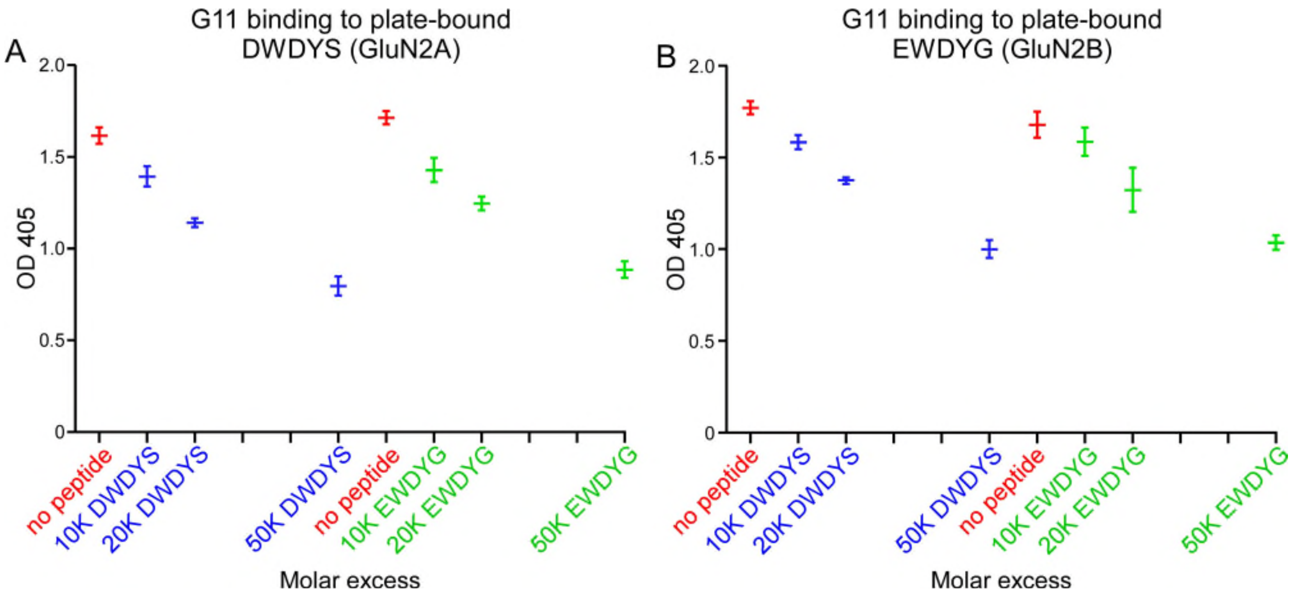

Supplementary Figure 1. Competitive ELISAs for DNRAb binding to soluble GluN2A or GluN2B epitopes (relates to Figures 3B-3E).
(A & B) Optical density (OD 405) values (mean ± SEM), measured by competitive ELISAs, for displacement of human DNRAb (G11) from plate-bound **(A)** DWDYS pentapeptide (GluN2A-specific) or **(B)** EWDYG (GluN2B-specific) by molar excess concentrations of the same soluble pentapeptides (n = 4 for each treatment condition).

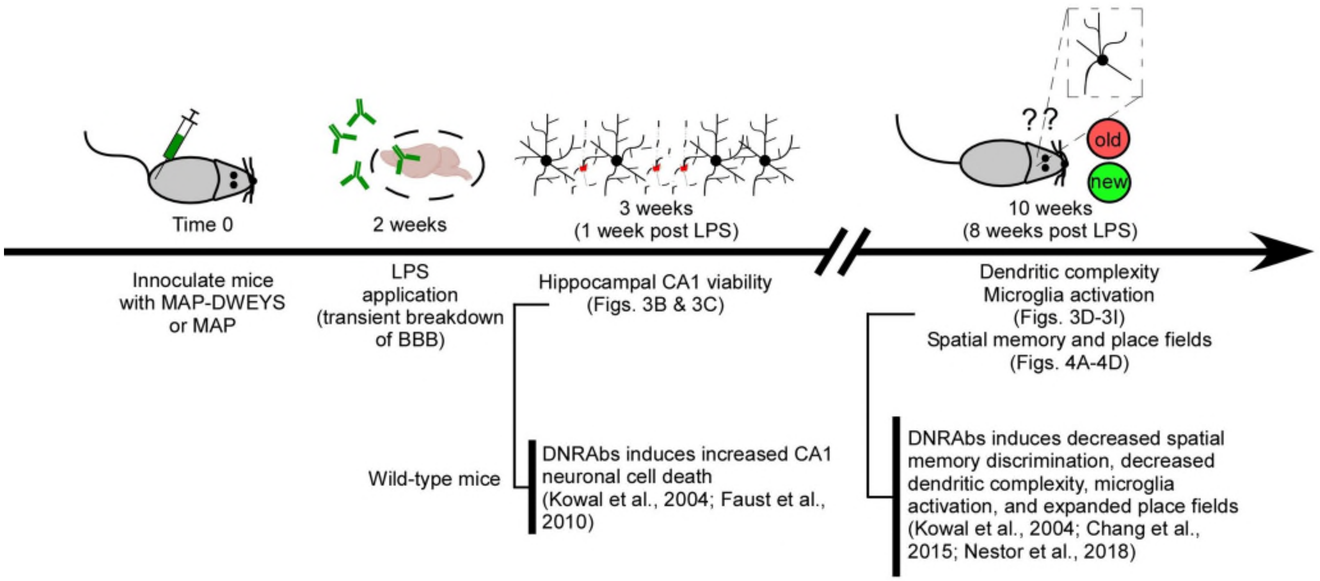

Supplementary Figure 2. Diagram of the experimental protocol used for the NPSLE mouse model (relates to Figures 4 & 5).

Female mice (6 – 8 weeks old) were immunized either with the DWEYS decapeptide multimerized on a polylysine backbone (MAP-DWEYS) or with the polylysine backbone alone (MAP). DWEYS is a mimetope of DNA and homologous to a sequence within the GluN2A and GluN2B amino-terminal domain. Immunization of wildtype mice with MAP-DWEYS induces production of DNRAbs (DNRAb+ mice) whereas MAP alone (control) does not[1]. Booster immunizations were applied two and four weeks after the first immunization. Time 0 is the final immunization with MAP-DWEYS or MAP (four weeks after initial). Two weeks after the final immunization, mice were given lipopolysaccharide (LPS) to allow transient access of antibodies to the hippocampus[2, 3]. One week after the LPS treatment subsets of mice were sacrificed for histological characterization of CA1 pyramidal cell viability (Figs. 4B & 4C)[2, 4]. At this time point following LPS treatment, DNRAbs are still present in the hippocampus[2, 5]. Two weeks post-LPS, DNRAb levels are not detectable[5]. Eight weeks post LPS, subsets of animals were tested for hippocampal anatomy (CA1 dendritic complexity and microglia activation are altered in treated wild-type mice[3] (Figs. 4D-4I). Other mice were tested for spatial memory test and implanted with tetrodes to determine place field size (Figs 5A-5D).

Use of LPS to permealize BBB. LPS by itself produces a systemic inflammatory response and neuroinflammatory effects leading to neuronal death and microglia activation[6, 7, 8]. However, for all of our *in vivo* experiments, we only make comparisons between DNRAb+ (experimental) mice and DNARb- (control) mice, with both of these groups being treated with LPS. In addition, LPS permealization localizes DNRAbs more in the hippocampus and related structures than in other brain regions[2, 9]. The major region of action of DNRAbs in patients is the hippocampus and related structures[10, 11].

SUPPLEMENTARY METHODS

Immunization

Immunization with MAP-core and MAP-DWEYS peptide was performed according to an established immunization protocol for DNRAb generation[2, 3, 5]. Female mice aged 6–8 weeks were first immunized with the multi-antigen, given in Complete Freund's Adjuvant (Becton, Dickinson, and Company, 263810), with two boosters at 2 and 4 weeks in Incomplete Freund's Adjuvant (Becton, Dickinson, and Company, 263910). At 2 weeks following the second booster, mice received two intraperitoneal injections of LPS (6 mg/kg; Millipore-Sigma, L4524) 48 h apart administered with a 500 μl intraperitoneal injection of sterile saline.

In vitro cell culture and transfection

Human embryonic kidney 293 (HEK293) cells were grown in Dulbecco's modified Eagle's medium (DMEM), supplemented with 10% FBS, for 24 h before transfection. Human NMDAR-encoding cDNA constructs (Table 1) were co-transfected into HEK293 cells along with a separate peGFP-Cl construct at a ratio of 4:4:1 (N1:N2:eGFP) for macroscopic recordings, and at a ratio of 4:1.5:1 for single channel recordings using X-tremeGene HP (Roche, 06-366). Triheteromeric NMDAR-expressing constructs (Table 1), all derived from rat, were a gift from K. Hansen and transfection with these constructs were performed with Ca^{2+} precipitation[12]. HEK293 cells were bathed in medium containing the GluN2 competitive antagonist DL-2-amino-5-phosphopentanoic acid (APV, 100 µM, Tocris), magnesium (100 µM), and the GluN1 competitive antagonist 6,7-dichlorokynuric acid (DCKA, 100 µM, Tocris). All experiments were performed 24–48 h post-transfection. Point mutations were introduced in the various GluN2A subunits using site-directed mutagenesis (SDM)[13].

Table 1. Plasmid constructs used for heterologous expression of NMDARs in HEK293 cells.

Construct	Species	Description
pCI-neo-hGluN1a	Human	Wild-type receptor subunit
pCI-neo-hGluN2A	Human	Wild-type receptor subunit
pCI-neo-hGluN2B	Human	Wild-type receptor subunit
pCI-neo-2xATG-rGluN1a-GFP	Rat	Wild-type receptor subunit with bicistronic expression of eGFP (gift from K. Hansen)
pCI-neo-rGluN2A-C1-L4	Rat	Tri-heteromeric modified subunit (Hansen et al., 2014)
pCI-neo-rGluN2A-C2-L4	Rat	Tri-heteromeric modified subunit
pCI-neo-rGluN2B-AC1-L4	Rat	Tri-heteromeric modified subunit
pCI-neo-rGluN2B-AC2-L4	Rat	Tri-heteromeric modified subunit

In vitro macroscopic recordings

Macroscopic currents in the whole-cell mode were recorded at room temperature (20–23°C) using an EPC-10 amplifier with PatchMaster software (version v2x90.2, HEKA), digitized at 10 kHz and low-pass filtered at 2.9 kHz (−3 dB) using an 8 pole low pass Bessel filter[14]. Patch microelectrodes were filled with an intracellular solution (mM): 140 KCl, 10 HEPES, 1 BAPTA, 4 Mg^{2+}-ATP, 0.3 Na^{+}-GTP, pH 7.3 (KOH), 297 mOsm (sucrose). Our standard extracellular solution consisted of (mM): 150 NaCl, 2.5 KCl, and 10 HEPES, pH 7.2 (NaOH). Ca^{2+} was omitted from the extracellular solution to prevent rundown over time. Pipettes had resistances of 2–6 MΩ when filled with the pipette solution and measured in the standard Na^{+} external solution. We did not use series resistance compensation nor did we correct for junction potentials. Currents were measured within 10 min of going whole-cell.

External solutions were applied using a piezo-driven double barrel application system. Prior to use, we incubated the application system with 2% BSA (1xPBS) for 2 h at 21–24°C to minimize non-specific

antibody binding. For agonist application, one barrel contained the external solution 0.1 mM glycine, whereas the other barrel contained both 0.1 mM glycine and 1 mM glutamate. For display, NMDAR currents were digitally re-filtered at 500 Hz and resampled at 1 kHz using IgorPro (version 7, WaveMetrics).

In vitro single channel recordings

Single channel currents were recorded in the on-cell configuration at 20–23°C using an integrating patch clamp amplifier (Axopatch 200B, Molecular Devices), analog filtered at 10 kHz (four-pole Bessel filter), and digitized between 25 and 50 kHz (ITC-16 interfaced with PatchMaster, (version v2x90.2, HEKA)[15]. Data were transferred to QuB (version 2.0.0.30, SUNY at Buffalo) for analysis.

Patch pipettes (thick-wall, borosilicate, Sutter Instruments) were pulled and fire-polished achieving resistances between 10 and 20 MΩ when measured in the bath. At −100 mV, seal resistance ranged between 2 and 20 GΩ. For cell-attached recordings, patch pipettes were filled with the standard bath solution as well as 1 mM glutamate and 0.1 mM glycine. 0.05 mM EDTA was added to minimize gating effects of divalent cations. Inward currents were elicited by applying a pipette potential of +100 mV. Recordings of hGluN1/hGluN2A or hGluN1/hGluN2B, either in the presence of absence of DNRAbs, consisted of long clusters of activity separated by seconds-long periods of inactivity, simplifying detection of several channels in the patch. For these recordings the relatively high equilibrium open probability (eq. Po) and duration of recordings (≈10,000 to 180,000 per recording) indicated that we were recording from single-channel patches.

For DNRAbs at 10 μg/ml, our standard bath (pipette) solution consisted of 150 mM NaCl, 10 mM HEPES (pH 7.2, NaOH). For DNRAbs at 100 μg/ml, our standard bath had a decreased concentration of NaCl to 140 mM to compensate for the osmotic force generated by the antibodies in solution.

Immunocytochemistry

HEK293T cells were used for immunocytochemistry (ICC). Transfections were performed in the same manner as described above with X-tremeGene HP (Roche, 06-366), without GFP co-transfection. Cells were fixed with 4% paraformaldehyde 48 h post-transfection, washed 3 times with 1xPBS, and then blocked with 2% bovine serum albumin (BSA) (Sigma, A9647) at room temperature. Incubation of primary antibody, either IgG control B1 (20 µg/mL) or human DNRAb, G11 (20 µg/mL) diluted in 2% BSA was done overnight at 4°C. Following 3 washes with 1xPBS, labeling was performed with secondary goat anti-human Alexa 488-conjugated antibodies (1:1000, ThermoFisher, A-11013) at room temperature. Coverslips were mounted with ProLong® Diamond Anti-fade mountant w/DAPI (ThermoFisher, P36962), and imaged with an Olympus FV-1000 confocal microscope with FluoView software (FV10-ASW, version 4.02, Olympus). Five fields (212 x 212 µm, 60x oil) were taken per coverslip. Mean corrected fluorescence of fields were determined on ImageJ with spectral deconvolution plugin (Seth Gammon, Washington University at St. Louis)[16, 17] and ROI background correction.

For primary hippocampal neurons used for ICC, cultures were made according to an established postnatal culture protocol with minor modifications [18]. Glia and astrocyte harvested from P2 mice were seeded at 10-14 days prior to dissection[19]. On day of dissection, glia/astrocyte cultures had media replaced with plating media, consisting of 10% (v/v) Fetal Bovine Serum (FBS) (ThermoFisher, 16140071), 0.45% (w/v) glucose, 1 mM sodium pyruvate, 2 mM glutamine, 100 U/mL penicillin/streptomycin in Basal Medium Eagle (ThermoFisher, 21010046). Hippocampi from P0-P1 C57/BL6 mice pips were dissected in ice-cold dissection media, consisting of 0.11 mg/mL sodium pyruvate, 0.1% glucose, 10 mM HEPES in HBSS, Ca^{2+} and Mg^{2+}-free (ThermoFisher, 14175095) followed by 0.25% (v/v) trypsin-EDTA digestion for 20 min at 37°C. DNase I (Sigma, DN25) was added shortly after, and incubated for 5 min at RT. Following 2 washes with plating media, tissue was triturated with an FBS-coated pipette tip 10–12 times. Cells in suspension were counted with a

hemocytometer, with approximately 150,000 cells added to 18 mm glass coverslips coated with poly-D-lysine in 12-well plates. Cultures were incubated in plating medium for 2 h at 37°C to allow for cells to adhere to coverslip. Coverslips were then flipped onto glia/astrocyte cultures, and cells were allowed to grow overnight in plating media, then switched with maintenance medium, consisting of 2% B-27 supplement (ThermoFisher, 17504044), 2 mM glutamine, 100 U/mL penicillin/streptomycin. On DIV3, 5 µM of cytosine arabinoside (Sigma, C1768) was added to cultures to inhibit mitosis of non-neuronal cells. Thereafter, half of maintenance media was replaced every 3 days until pharmacological treatment at DIV14.

Pharmacological treatment of primary hippocampal neurons occurred at DIV14. Final concentrations of antagonists were 3 µM: TCN-201 (AdooQ Bioscience, A11947), MPX-004 (Alomone Labs, M280), and ifenprodil (Sigma, I2892). These were added along with the DNRAb (G11) at 10 µg/mL, in maintenance media to neurons. DMSO was used as vehicle control for both control (B1) and DNRAb (G11) conditions.

ICC was performed 24 h post treatment on DIV15. Coverslips were washed with 1xPBS 3 times and then incubated in 4% paraformaldehyde(v/v)/4% sucrose (w/v) for 15 min. Coverslips were then washed again with 1xPBS 3 times and were blocked/permeabilized with antibody blocking solution, consisting of 2% (w/v) BSA, 0.25% (v/v) Triton-X100 in 1xPBS, for 60 min at RT. Coverslips were then incubated in primary antibodies, rabbit anti-activated caspase-3 (1:250, Cell Signaling, 9661), and mouse anti-β tubulin III (Tubb3) (1:400, Millipore-Sigma, MAB1637), both in antibody blocking solution overnight in 4°C. Coverslips were then washed with 1xPBS 3 times, and incubated in fluorescence-conjugated secondary antibodies, Goat anti-rabbit Alexa-647 (1:1000, ThermoFisher, A21245) and Goat anti-mouse Alexa-488(1:1000, ThermoFisher, A21121) for 1 h at RT. Coverslips were then mounted and imaged as described above with Olympus confocal. Number of activated caspase-3 neurons were averaged by 5 fields (212 x 212 µm, 60x oil) per biological replicate. Quantification and image analysis were done on ImageJ by researcher blinded to treatment conditions.

ELISA

Peptides were coated on COSTAR plates (Corning, CLS3690) at 20 µg/ml, 25 µl/well in 0.1 M $NaHCO_3$ pH 8.6, at 4°C, overnight. The plate was washed once with 1x PBS containing 0.05 % Tween 100 (PBS-T) and blocked with 1% BSA/PBS, 50 µl/well, for 1 hr at 37^0C. G11 antibody was used at 7.5 µg/ml which was on the linear range of binding curve established prior to the inhibition. Antibody was pre-incubated with a wide range of the inhibitory peptide in 1% BSA/PBS for 1 hr at RT and added to each well. The assay was performed twice with triplicates measurements. The well without the inhibitor peptide served as positive control. After incubation at 37°C for 1 hr, the plate was washed 6 times with PBS-T buffer. Goat anti-human secondary antibody labeled with alkaline phosphatase (IgG-AP) (SouthernBiotech, 2040-40) was added at 1:1000 dilution in 25 µl/well of 1% BSA/PBS followed by incubation at 37°C for 1 hr and by washing with PBS-T buffer. The ELISA was developed using AP substrate (Millipore-Sigma), in 50 µl/well of a buffer containing $MgCl_2$ (1 mM final), Na_2CO_3 (25 mM final) and $NaHCO_3$ (2.5 mM final). The reading was done at 405 µm using a multilabel counter from Perkin-Elmer.

Neuronal Staining

Mice were anesthetized with 100 µl of Euthasol (Virbac) prior to perfusion with 0.9% sodium chloride, 0.5% sodium nitrite, and 0.1% heparin, followed by 4% paraformaldehyde (PFA) in 0.1 M phosphate buffer (PB) as before[3].

Cresyl violet staining. Brains were extracted, fixed in 4% PFA for 2 h and transferred to 30% sucrose, blocked and sliced at 40 µm. Brains were blocked in a coronal stainless-steel template and a 4 mm slab of tissue was cut from approximately Bregma -0.94 mm with a microtome. Tissue was sampled at a thickness of 40 µm with a periodicity of one in four coronal sections over the next 1600 to 1900 µm.

Sections were mounted, dehydrated, rehydrated and stained in cresyl violet for 3 min. Sections were dried, dehydrated, cleared (Histoclear II) and coverslipped with Permount® (ThermoFisher) prior to imaging on an AxiophotZ1 microscope (Zeiss). Sections were imaged in 100x oil, and we focused on the soma of the stratum pyramidale around which a tiled Z-stacked (0.5 µm steps) image was generated. Stereology was performed with the sampling frame set such that an individual frame captures 2–5 targets in the photomicrograph[2, 3, 5]:. Then optical dissectors are created by manipulating the level of the Z-stack, and nuclei within neurons in focus are counted as long as they are not in contact with the left or bottom part of a frame[20]. Quantitation of CA1 neurons in image stacks was performed with the Stereo Investigator suite in Neurolucida 360 (MBF Bioscience) with researcher blinded to treatment conditions. Neuron numbers in Fig. 4C represent the total number of cell bodies captured in the systematic random sampling frame as it is placed on consecutive sections or “runs”. There were 3 animals in each group, with 8 runs for each animal.

Golgi stain for neuronal Sholl analysis. Brains were processed using FD Rapid Golgi Stain kit (FD NeuroTechnologies), a silver staining method to visualize entire neurons[21]. Brains were extracted and submerged in the impregnation solution (Solution A + Solution B, 1:1) for 2 weeks. Impregnation solution was changed after the first 24 h. Brains were then cryoprotected in Solution C for 72 h (room temperature, dark). Solution C was exchanged after the first 24 h. After 14 days, brains were sectioned at 100 µm on a cryostat (-21°C) that permit identification of internal anatomic landmarks to signal sample start and stop, with periodicity of one in four coronal sections across ~1200 µm (Bregma -0.94 to -2.2mm for the dorsal hippocampus) of brain with at least 5 different sections being sample per animal. Sections were mounted, dried (room temperature, dark), silver nitrate stained (Solution D + Solution E, 1:1), rinsed, dehydrated in EtOH, cleared in xylene, and coverslipped. Tissue was imaged on an AxioImager Z1 microscope (Zeiss) with ZEN Blue (version 2.0, Zeiss) software in 40x oil using tiling and Z-stack (2 µm steps). There were 4 animals in each group and 10–20 neurons were analyzed for each animal as shown in Fig. 4D&E. Images were then analyzed using Neurolucida 360 (MBF Bioscience) dendritic tracing for Sholl analysis at 10 µm shells from the soma by researcher blinded to treatment conditions[3, 5].

Immunohistochemistry

For immunohistochemistry, brains were extracted and sectioned as performed for cresyl violet staining above. Sections were washed (0.1 M phosphate buffered saline, PBS), permeabilized (0.2% Triton X-100 in 1% BSA in 0.1 M PBS), blocked (1% BSA in 0.1 M PBS for 60 minutes), and stained with primary antibody overnight at 4°C in 1% BSA. Primary antibodies were rabbit anti-Iba1 (1:500, Wako Chemicals, 019-1 9741), rat anti-CD68 (1:500, Bio-Rad, mca1957). On the following day, samples were washed (0.1 M PBS), incubated with secondary antibody (0.1 M PB for 45 min). Secondary antibodies included Donkey anti-rat Alexa Fluor 488 (1:400, Life Technologies, A21208), Chicken anti-rabbit Alexa Fluor 594 (1:300, Life Technologies, A21442). Sections underwent additional washes and incubation in DAPI (0.5 µg/mL in 0.1 M PB) and mounted with Cytoseal 60 (ThermoFisher) and cover-slipped.

Tissue was imaged on an AxioImager Z1 microscope or LSM 880 confocal microscope using Airy scan parameters (Zeiss). Microglia and colocalization with CD68 were quantified using ZEN Blue (version 2.0, Zeiss) software[3, 22]. CD68 score was graded as before: “0”, no or scarce expression; “1”, punctate expression; “2”, aggregate or punctate expression all over cell [3]. Microglia were quantitated per 106.4 μm^2 box that was placed in 6 regions of interest across the stratum radiatum of CA1 using identical Airy scan acquisition parameters (40x oil, Airy = 2X, Z-stack = 10). Images of individual microglia were transferred to Neurolucida 360 (MBF Bioscience) for quantitation. We analyzed 3 tissue sections; the periodicity was one in four 40 µm sections across the dorsal CA1 hippocampus, there were 3 animals in each group. All raw measurements were analyzed by a researcher blinded to treatment conditions and compiled for cumulative probability distributions and analyzed by Kolmogorov-Smirnov

non-parametric statistics. The mean results for each Sholl dimension from the soma were plotted for each group and displayed in 5 μm concentric circle increments.

Behavioral assessment and *in vivo* electrophysiology

Mice used for these procedures lived on a reverse light-dark cycle (lights off at 09:00 h, lights on at 21:00 h), for at least 10 days before testing started, with *ad libitum* access to chow and water. All behavioral experiments occurred between 10:00 h and 18:00 h, which corresponded to the dark phase of the circadian cycle, when the rodents were naturally active. Animals were handled for 15 min per day for 3 days before they were tested behaviorally. The object place memory (OPM) task was performed as described [3, 5]. The apparatus consisted of a square chamber (40 cm on the side, 40 cm high), with the walls painted grey. Researchers were blinded to animal's treatment group during behavior assessment. Animals were familiarized to the empty chamber (3 sessions of 15 min each). For OPM testing, mice underwent the following sequence: empty chamber (10 min), home cage (10 min), sample phase in which the chamber had two objects located at the center of the NW and NE sectors (5 min), home cage (10 min), choice phase in which the chamber had the same objects but one of them was moved from NE to the center of the SE sector (5 min). The discrimination ratio was calculated during the choice phase by dividing time spent exploring the moved object minus the time spent exploring the static object by the time exploring the objects combined [5]. Data were collected and analyzed using EthoVision XT (version 11, Noldus Information Technologies, Leesburg, VA) which automatically detected the contours of the animal from a live video feed, discriminated it from the background, and tracked activity with a 3-point detection algorithm (video rate, 30 frames per second).

The analysis of place cells in the dorsal CA1 region of the hippocampus: a mouse was anesthetized with 0.25% isoflorane and implanted with a 16 channel multi-electrode array containing 4 tetrodes[4, 5]. After recovery, single unit firing was recorded using Cheetah software (version 5, Neuralynx) while the animal explored a square chamber (40 cm on the side) in a schedule of 4 exploration runs (15 min) separated by 3 rest sessions (5 min) in the home cage. Recordings were repeated over 2 consecutive days. Acquired data were analyzed using Spike2 (version 8, Cambridge Electronic Design), NeuroExplorer (version 5, Nex Technologies), and MATLAB (version 9.2 R2017a, MathWorks).

SUPPLEMENTARY INFORMATION REFERENCES

1. Putterman C, Diamond B. Immunization with a peptide surrogate for double-stranded DNA (dsDNA) induces autoantibody production and renal immunoglobulin deposition. *J Exp Med* **188**, 29-38 (1998).

2. Kowal C*, et al.* Cognition and immunity; antibody impairs memory. *Immunity* **21**, 179-188 (2004).

3. Nestor J*, et al.* Lupus antibodies induce behavioral changes mediated by microglia and blocked by ACE inhibitors. *J Exp Med*, (2018).

4. Faust TW*, et al.* Neurotoxic lupus autoantibodies alter brain function through two distinct mechanisms. *Proc Natl Acad Sci U S A* **107**, 18569-18574 (2010).

5. Chang EH*, et al.* Selective Impairment of Spatial Cognition Caused by Autoantibodies to the N-Methyl-D-Aspartate Receptor. *EBioMedicine* **2**, 755-764 (2015).

6. Sternberg EM. Neural-immune interactions in health and disease. *J Clin Invest* **100**, 2641-2647 (1997).

7. Nolan Y, Vereker E, Lynch AM, Lynch MA. Evidence that lipopolysaccharide-induced cell death is mediated by accumulation of reactive oxygen species and activation of p38 in rat cortex and hippocampus. *Exp Neurol* **184**, 794-804 (2003).

8. Vereker E, Campbell V, Roche E, McEntee E, Lynch MA. Lipopolysaccharide inhibits long term potentiation in the rat dentate gyrus by activating caspase-1. *J Biol Chem* **275**, 26252-26258 (2000).

9. Huerta PT, Kowal C, DeGiorgio LA, Volpe BT, Diamond B. Immunity and behavior: antibodies alter emotion. *Proc Natl Acad Sci U S A* **103**, 678-683 (2006).

10. Appenzeller S, Carnevalle AD, Li LM, Costallat LT, Cendes F. Hippocampal atrophy in systemic lupus erythematosus. *Ann Rheum Dis* **65**, 1585-1589 (2006).

11. Mackay M*, et al.* Metabolic and microstructural alterations in the SLE brain correlate with cognitive impairment. *JCI Insight* **4**, (2019).

12. Hansen KB, Ogden KK, Yuan H, Traynelis SF. Distinct functional and pharmacological properties of Triheteromeric GluN1/GluN2A/GluN2B NMDA receptors. *Neuron* **81**, 1084-1096 (2014).

13. Kazi R, Dai J, Sweeney C, Zhou HX, Wollmuth LP. Mechanical coupling maintains the fidelity of NMDA receptor-mediated currents. *Nat Neurosci* **17**, 914-922 (2014).

14. Yelshansky MV, Sobolevsky AI, Jatzke C, Wollmuth LP. Block of AMPA receptor desensitization by a point mutation outside the ligand-binding domain. *J Neurosci* **24**, 4728-4736 (2004).

15. Talukder I, Wollmuth LP. Local constraints in either the GluN1 or GluN2 subunit equally impair NMDA receptor pore opening. *J Gen Physiol* **138**, 179-194 (2011).

16. Gammon ST, Leevy WM, Gross S, Gokel GW, Piwnica-Worms D. Spectral unmixing of multicolored bioluminescence emitted from heterogeneous biological sources. *Anal Chem* **78**, 1520-1527 (2006).

17. Zurek-Biesiada D, Kedracka-Krok S, Dobrucki JW. UV-activated conversion of Hoechst 33258, DAPI, and Vybrant DyeCycle fluorescent dyes into blue-excited, green-emitting protonated forms. *Cytometry A* **83**, 441-451 (2013).

18. Beaudoin GM, 3rd, *et al.* Culturing pyramidal neurons from the early postnatal mouse hippocampus and cortex. *Nat Protoc* **7**, 1741-1754 (2012).

19. Kaech S, Banker G. Culturing hippocampal neurons. *Nat Protoc* **1**, 2406-2415 (2006).

20. Gundersen HJ. Stereology of arbitrary particles. A review of unbiased number and size estimators and the presentation of some new ones, in memory of William R. Thompson. *J Microsc* **143**, 3-45 (1986).

21. Glaser EM, Van der Loos H. Analysis of thick brain sections by obverse-reverse computer microscopy: application of a new, high clarity Golgi-Nissl stain. *J Neurosci Methods* **4**, 117-125 (1981).

22. Schafer DP, *et al.* Microglia sculpt postnatal neural circuits in an activity and complement-dependent manner. *Neuron* **74**, 691-705 (2012).
