# Supplementary material for: Lupus autoantibodies act as positive allosteric modulators at GluN2A-containing NMDA receptors and impair spatial memory
Source: Nat Commun. 2020 Mar 16;11:1403. doi: 10.1038/s41467-020-15224-w (PMC7075964; doi:10.1038/s41467-020-15224-w)
Supplement: Supplementary file 2 — Reporting summary [file 41467_2020_15224_MOESM2_ESM.pdf]

natureresearch

Corresponding author(s): Betty Diamond, Lonnie Wollmuth

Last updated by author(s): 02/06/2020

Reporting Summary

Nature Research wishes to improve the reproducibility of the work that we publish. This form provides structure for consistency and transparency in reporting. For further information on Nature Research policies, see Authors & Referees and the Editorial Policy Checklist .

Statistics

For all statistical analyses, confirm that the following items are present in the figure legend, table legend, main text, or Methods section.

n/a	Confirmed	
☐	☒	The exact sample size (*n*) for each experimental group/condition, given as a discrete number and unit of measurement
☐	☒	A statement on whether measurements were taken from distinct samples or whether the same sample was measured repeatedly
☐	☒	The statistical test(s) used AND whether they are one- or two-sided<br>*Only common tests should be described solely by name; describe more complex techniques in the Methods section.*
☒	☐	A description of all covariates tested
☐	☒	A description of any assumptions or corrections, such as tests of normality and adjustment for multiple comparisons
☐	☒	A full description of the statistical parameters including central tendency (e.g. means) or other basic estimates (e.g. regression coefficient) AND variation (e.g. standard deviation) or associated estimates of uncertainty (e.g. confidence intervals)
☐	☒	For null hypothesis testing, the test statistic (e.g. *F*, *t*, *r*) with confidence intervals, effect sizes, degrees of freedom and *P* value noted<br>*Give P values as exact values whenever suitable.*
☒	☐	For Bayesian analysis, information on the choice of priors and Markov chain Monte Carlo settings
☒	☐	For hierarchical and complex designs, identification of the appropriate level for tests and full reporting of outcomes
☒	☐	Estimates of effect sizes (e.g. Cohen's *d*, Pearson's *r*), indicating how they were calculated

Our web collection on statistics for biologists contains articles on many of the points above.

Software and code

Policy information about availability of computer code

Data collection

Commercial software used for collection is detailed in Methods and Supplementary Methods file. The following software used for data collection are:
Patchmaster (version v2x90.2, HEKA)
FluoView (FV10-ASW, version 4.02, Olympus)
ZEN Blue (version 2.0, Zeiss)
EthoVision XT (version 11, Noldus)
Cheetah (version 5, Neuralynx)

Data analysis	Commercial software used for analysis is detailed in Methods and Supplementary Methods file. The following software used for data analysis are:<br>QuB (version 2.0.0.30, SUNY at Buffalo)<br>Igor Pro (version 7, WaveMetrics)<br>Neurolucida 360 (MBF Bioscience)<br>EthoVision XT (version 11, Noldus)<br>Spike2 (version 8, Cambridge Electronic Design)<br>NeuroExplorer (version 5, Nex Technologies)<br>MATLAB (version 9.2, MathWorks)<br>Origin Pro (version 9, Origin Lab)<br>MiniTab (version 19, Trialware)<br>ImageJ<br>Microsoft Excel

For manuscripts utilizing custom algorithms or software that are central to the research but not yet described in published literature, software must be made available to editors/reviewers. We strongly encourage code deposition in a community repository (e.g. GitHub). See the Nature Research guidelines for submitting code & software for further information.

Data

Policy information about availability of data

All manuscripts must include a data availability statement. This statement should provide the following information, where applicable:

- Accession codes, unique identifiers, or web links for publicly available datasets
- A list of figures that have associated raw data
- A description of any restrictions on data availability

The datasets generated and analyzed during the current study are available from the corresponding authors on reasonable request. The source data underlying Figures 1-5 has been provided as a Source Data file in Microsoft Excel format.

Field-specific reporting

Please select the one below that is the best fit for your research. If you are not sure, read the appropriate sections before making your selection.

[x] Life sciences [ ] Behavioural & social sciences [ ] Ecological, evolutionary & environmental sciences

For a reference copy of the document with all sections, see nature.com/documents/nr-reporting-summary-flat.pdf

Life sciences study design

All studies must disclose on these points even when the disclosure is negative.

Sample size	We do not use any statistical tests to choose sample size a priori. The sample was chosen from previous publications with similar techniques used (see Nestor et al., 2018, J Exp Med; Amin et al., 2018, Nat Commun). This is detailed in Methods section (pp.23).
Data exclusions	No data were excluded.
Replication	Samples are replicated at least an n of 3 for all experiments. All attempts at replication were successful.
Randomization	Not applicable as treatments would have to be applied on the correct genotype or construct.
Blinding	Investigators were blinded described in Supplementary Methods. Researchers were blinded to treatment conditions during analysis for the Method subheadings: Immunocytochemistry, Neuronal Staining, and Immunohistochemistry. Researchers were blinded to group allocation during data collection for the Method subheadings: Behavior Assessment & In vivo electrophysiology.

Reporting for specific materials, systems and methods

We require information from authors about some types of materials, experimental systems and methods used in many studies. Here, indicate whether each material, system or method listed is relevant to your study. If you are not sure if a list item applies to your research, read the appropriate section before selecting a response.

Materials & experimental systems

n/a	Involved in the study	
☐	☒	Antibodies
☐	☒	Eukaryotic cell lines
☒	☐	Palaeontology
☐	☒	Animals and other organisms
☒	☐	Human research participants
☒	☐	Clinical data

Methods

n/a	Involved in the study	
☒	☐	ChIP-seq
☒	☐	Flow cytometry
☒	☐	MRI-based neuroimaging

Antibodies

Antibodies used

All antibodies listed in Methods (pp.17-22), with manufacturers and catalogue numbers elaborated in Supplemental Methods.
Antibody list:
Immunocytochemistry
G11 or B1 (see below)
Goat anti-human Alexa-488 (ThermoFisher, A-11013)
Rabbit anti-activated Caspase-3 (Cell Signaling, 9661)
Mouse anti-beta tubulin III (Millipore-Sigma, MAB1637)
Goat anti-rabbit Alexa-647 (ThermoFisher, A21245)
Goat anti-mouse Alexa-488 (ThermoFisher, A21121)

ELISA
Goat anti-human Alkaline Phosphatase (SouthernBiotech, 2040-40)

Immunohistochemistry
Rabbit anti-Iba1 (Wako Chemicals, 019-1 9741)
Rat anti-CD68 (Bio-Rad, mca1957)
Chicken anti-rabbit Alexa-594 (Life Technologies, A21442)
Donkey anti-rat Alexa-488 (Life Technologies, A21208)

The B1 and G11 antibody source are from a female SLE patient that has been previously validated (see Zhang et al., 2009, J Autoimmunity; Faust et al., 2010, PNAS).

Validation

The B1 and G11 antibody has been previously validated (see Zhang et al., 2009, J Autoimmunity; Faust et al., 2010, PNAS) and has been elaborated on in Methods (pp.17) and in Supplementary Material (pp. 5).

All antibodies used in immunocytochemistry and immunohistochemistry are from commercial sources as listed in Methods (pp.19-22). Please see manufacturer's link for validation of antibody as well as previous publications that use the antibody:

Immunocytochemistry
Goat anti-human Alexa-488 (ThermoFisher, A-11013)
https://www.thermofisher.com/antibody/product/Goat-anti-Human-IgG-H-L-Cross-Adsorbed-Secondary-Antibody-Polyclonal/A-11013
Rabbit anti-activated Caspase-3 (Cell Signaling, 9661)
https://www.cellsignal.com/products/primary-antibodies/cleaved-caspase-3-asp175-antibody/9661
Mouse anti-beta tubulin III (Millipore-Sigma, MAB1637)
https://www.emdmillipore.com/US/en/product/Anti-Tubulin-Antibody-beta-III-isoform-CT-clone-TU-20-Similar-to-TUJ1,MM_NF-MAB1637
Goat anti-rabbit Alexa-647 (ThermoFisher, A21245)
https://www.thermofisher.com/antibody/product/Goat-anti-Rabbit-IgG-H-L-Highly-Cross-Adsorbed-Secondary-Antibody-Polyclonal/A-21245
Goat anti-mouse Alexa-488 (ThermoFisher, A21121)
https://www.thermofisher.com/antibody/product/Goat-anti-Mouse-IgG1-Cross-Adsorbed-Secondary-Antibody-Polyclonal/A-21121

ELISA
Goat anti-human Alkaline Phosphatase (SouthernBiotech, 2040-40)
https://www.southernbiotech.com/?catno=2040-04&type=Polyclonal#&panel1-1&panel2-1

Immunohistochemistry
Rabbit anti-Iba1 (Wako Chemicals, 019-1 9741)
https://labchem-wako.fujifilm.com/us/product/detail/W01W0101-1974.html
Rat anti-CD68 (Bio-Rad, mca1957)

https://www.bio-rad-antibodies.com/monoclonal/mouse-cd68-antibody-fa-11-mca1957.html?f=purified
Chicken anti-rabbit Alexa-594 (Life Technologies, A21442)
https://www.thermofisher.com/antibody/product/Chicken-anti-Rabbit-IgG-H-L-Cross-Adsorbed-Secondary-Antibody-Polyclonal/A-21442
Donkey anti-rat Alexa-488 (Life Technologies, A21208)
https://www.thermofisher.com/antibody/product/Donkey-anti-Rat-IgG-H-L-Highly-Cross-Adsorbed-Secondary-Antibody-Polyclonal/A-21208

Eukaryotic cell lines

Policy information about cell lines

Cell line source(s)	HEK293 and HEK293T cell lines from the supplier, ATCC.
Authentication	None of the cell lines were authenticated.
Mycoplasma contamination	Yes, a PCR-based mycoplasma detection kit is regularly employed to detect mycoplasma in cell cultures. All PCR tests were negative for mycoplasma.
Commonly misidentified lines (See ICLAC register)	None

Animals and other organisms

Policy information about studies involving animals; ARRIVE guidelines recommended for reporting animal research

Laboratory animals	For behavior and in vivo electrophysiology, female mice (age 6-8 weeks at first immunization, strain: C57BL/6) were used with housing conditions mentioned in Methods (pp.17) and Supplementary Methods (pp.5) prior to experiments. For primary hippocampal cultures, unsexed p0/p1 mice pups (strain: C57BL/6) were sacrificed for cultures. Otherwise, mice used for management of the colony were housed in a laboratory animal facility with a 12/12 hr light/dark cycle and given food and water ad libitum.
Wild animals	No wild animals were used here in this manuscript.
Field-collected samples	No field collected samples used here in this manuscript.
Ethics oversight	IACUC from both the Feinstein Institute/Northwell Health and Stony Brook University approved protocols as mentioned in Methods (pp.17)

Note that full information on the approval of the study protocol must also be provided in the manuscript.
